# Minimal Residual Disease at First Achievement of Complete Remission Predicts Outcome in Adult Patients with Philadelphia Chromosome-Negative Acute Lymphoblastic Leukemia

**DOI:** 10.1371/journal.pone.0163599

**Published:** 2016-10-03

**Authors:** Mingming Zhang, Huarui Fu, Xiaoyu Lai, Yamin Tan, Weiyan Zheng, Jimin Shi, Yanmin Zhao, Maofang Lin, Jingsong He, Zhen Cai, Yi Luo, He Huang

**Affiliations:** Bone Marrow Transplantation Center, The First Affiliated Hospital, Zhejiang University School of Medicine, Hangzhou, People’s Republic of China; University of Sydney, AUSTRALIA

## Abstract

We evaluated the prognostic effect of minimal residual disease at first achievement of complete remission (MRD at CR1) in adult patients with Philadelphia chromosome-negative acute lymphoblastic leukemia (ALL). A total of 97 patients received treatment in our center between 2007 and 2012 were retrospectively reviewed in this study. Patients were divided into two arms according to the post-remission therapy (chemotherapy alone or allogeneic hematopoietic stem cell transplantation (allo-HSCT)) they received. MRD was detected by four-color flow cytometry. We chose 0.02% and 0.2% as the cut-off points of MRD at CR1 for risk stratification using receiver operating characteristic analysis. The 3-year overall survival (OS) and leukemia free survival (LFS) rates for the whole cohort were 46.2% and 40.5%. MRD at CR1 had a significantly negative correlation with survival in both arms. Three-year OS rates in the chemotherapy arm were 70.0%, 25.2%, 0% (P = 0.003) for low, intermediate, and high levels of MRD at CR1, respectively. Three-year OS rates in the transplant arm were 81.8%, 64.3%, 27.3% (P = 0.005) for low, intermediate, and high levels of MRD at CR1, respectively. Multivariate analysis confirmed that higher level of MRD at CR1 was a significant adverse factor for OS and LFS. Compared with chemotherapy alone, allo-HSCT significantly improved LFS rates in patients with intermediate (P = 0.005) and high (P = 0.022) levels of MRD at CR1, but not patients with low level of MRD at CR1 (P = 0.851). These results suggested that MRD at CR1 could strongly predict the outcome of adult ALL. Patients with intermediate and high levels of MRD at CR1 would benefit from allo-HSCT.

## Introduction

The outcome of adult ALL has improved over the past decades with overall survival (OS) reaching 35–50%.[1, 2] However, high relapse rate has always been the primary element to threaten the long-term survival, and is associated with a dismal survival rate of <10%.[3] Therefore, the identification of patients with high relapse risk and poor prognosis in complete remission becomes an important approach to improve the clinical outcome by early intensified treatment, including allogeneic hematopoietic stem cell transplantation (allo-HSCT).

Over the last decade, evaluation of minimal residual disease (MRD) has been widely used to identify patients with poor prognosis. MRD had been demonstrated to have the prognostic effect in adult ALL in several studies,[4–10] but the time points of MRD detection and the cut-offs of MRD were variable among these studies. Moreover, the prognostic effect of early MRD in allo-HSCT set had not been clearly defined,[8, 11] and the decision-making of post-remission therapy according to MRD level was still unclear at present.

In this study, we aimed to evaluate the prognostic effect of MRD levels at first achievement of complete remission (MRD at CR1) for adult patients with Philadelphia chromosome-negative (Ph-negative) ALL in chemotherapy and allo-HSCT arms, and further to discover subgroups that benefit from allo-HSCT based on the stratification by MRD at CR1.

## Methods

### Patients

A total of 97 consecutive patients diagnosed with Ph-negative ALL were retrospectively reviewed with the following inclusion criteria: (i) Ph-negative ALL (non-Burkitt type ALL) patients diagnosed between January 2007 and December 2012; (ii) age limited between 15 to 55 years old; (iii) reaching CR after one or more induction courses; (iv) keeping CR status at transplantation. Patients were classified as high risk if they met one of the following criteria at diagnosis: (i) cytogenetic abnormalities: t(4;11), or other 11q23 rearrangements, t(1;19), or complex karyotype (5 or more chromosomal abnormalities); (ii) high WBC count at diagnosis (≥30×10^9^/L in case of B-ALL; ≥100×10^9^/L in case of T-ALL); (iii) failure to achieve CR after first induction course. All other patients were classified as standard risk. Patients were divided into two arms according to the post-remission therapy (chemotherapy alone or allo-HSCT) they received. The clinical characteristics of the two arms were detailed in Table 1. The study was approved by ethics committee of the First Affiliated Hospital, College of Medicine, Zhejiang University in January 2014 and was conducted after the ethics approval. All of the patients and donors had signed and returned their written informed consent. Authors who were responsible for data collection had access to information that could identify individual participants during data collection

**Table 1:** Patient Characteristics.

Variables	Chemotherapy	Allo-HSCT	*P*
	No. (%)	No. (%)	
**Number of patients**	45	52	
**Age at diagnosis**			0.51
<35y	32 (71.1)	40 (76.9)	
≥35y	13 (28.9)	12 (23.1)	
**Male, sex**	31 (68.9)	29 (55.8)	0.19
**WBC count at diagosis**			0.50
<30×10^9^/L	26 (57.8)	31 (59.6)	
≥30×10^9^/L	18 (40.0)	16 (30.8)	
Unknown	1 (2.2)	5 (9.6)	
**B or T lineage**			0.59
B	35 (77.8)	38 (73.1)	
T	10 (22.2)	14 (26.9)	
**Cytogenetics**			0.81
Poor	4 (8.9)	3 (5.8)	
Others	37 (82.2)	46 (88.4)	
Unknown	4 (8.9)	3 (5.8)	
**Risk stratification**			0.77
Standard	21 (46.7)	26 (50.0)	
High	21 (46.7)	23 (44.2)	
Unknown	3 (6.6)	3 (5.8)	
**Induction courses before CR1**			0.63
1 course	38 (84.4)	44 (84.7)	
2 courses	6 (13.3)	5 (9.6)	
3 courses	1 (2.3)	2 (3.8)	
4 courses	0	1 (1.9)	
**Donor types**			NA
HLA-matched sibling	NA	13 (25.0)	
Unrelated	NA	22 (42.3)	
Haploidentical	NA	17 (32.7)	

HSCT: hematopoietic stem cell transplantation; WBC: white blood cell; NA: not applicable.

### Treatment procedure

The chemotherapy procedure included induction, consolidation, and maintenance chemotherapies. Induction regimens included VDCLP (vincristine 1.4 mg/m^2^/d, 2mg max, days 1, 8, 15, and 22; daunorubicin 45 mg/m^2^/d, days 1 to 3; cyclophosphamide 650 mg/m^2^/d, days 1, 8 and 15; prednisone 60 mg/m^2^/d, days 1 to 28; and L-asparaginase 10,000 U/m^2^/d, days 10 to 19), VDLP (vincristine 1.4 mg/m^2^/d, 2mg max, days 1, 8, 15, and 22; daunorubicin 45 mg/m^2^/d, days 1 to 3; prednisone 60 mg/m^2^/d, days 1 to 28; and L-asparaginase 10,000 U/m^2^/d, days 10 to 19), or VDCP (vincristine 1.4 mg/m^2^/d, 2mg max, days 1, 8, 15, and 22; daunorubicin 45 mg/m^2^/d, days 1 to 3; cyclophosphamide 650 mg/m^2^/d, days 1, 8 and 15; and prednisone 60 mg/m^2^/d, days 1 to 28). Two patients in the chemotherapy arm and two patients in the allo-HSCT arm received VDP (vincristine 1.4 mg/m^2^/d, 2mg max, days 1, 8, 15, and 22; daunorubicin 45 mg/m^2^/d, days 1 to 3; and prednisone 60 mg/m^2^/d, days 1 to 28) as induction. Consolidation regimens included hyper-CVAD (A) (cyclophosphamide 300 mg/m^2^ every 12hrs, days 1 to 3; doxorubicin 50 mg/m^2^, day 4; vincristine 2 mg/d, days 4 and 11; and dexamethasone 40 mg/d, days 1 to 4 and days 11 to 14), hyper-CVAD (B) (MTX 1 g/m^2^, day 1; and Ara-C 3 g/m^2^ every 12hrs, days 2 and 3), MTX+L-asparaginase (MTX 3 g/m^2^, day 1; and L-asparaginase 10,000 U/m^2^/d, days 2 to 8), CAM (cyclophosphamide 650 mg/m^2^/d, days 1, 15, and 29; Ara-C 75 mg/m^2^/d, days 2 to 5, days 9–12, days 16–19, and days 23–26; 6-thioguanine 60 mg/m^2^/d p.o., days 1 to 28), VMCP (vincristine 1.4 mg/m^2^/d, 2mg max, days 1, 8, 15, and 22; mitoxantrone 10 mg/m^2^/d, days 1 to 3; cyclophosphamide 650 mg/m^2^/d, days 1, 8 and 15; and prednisone 60 mg/m^2^/d, days 1 to 28), and also VDCLP as mentioned above, which were given in turn. Maintenance therapy continued for two years which consisted of vincristine 1.4 mg/m^2^ i.v. for 1 day every 3 months, prednisone 60 mg/m^2^ p.o. for 5 days every 3 months, 6-thioguanine 75 mg/m^2^ p.o. daily, and MTX 20 mg/m^2^ p.o. or i.v. weekly.

For patients in the allo-HSCT arm, high-resolution DNA typing was performed for HLA-A, -B, -C, -DRB1, and -DQB1. Myeloablative conditioning and graft-versus-host disease prophylaxis were given as described.[12]

All patients received prophylactic treatment of the central nervous system leukemia with intrathecal chemotherapy consisting of Ara-C and dexamethasone during remission.

### Investigation of minimal residual disease

Erythrocyte-lysed whole BM samples were used for immunophenotyping on the day of bone marrow aspiration. Antigen expression of blast cells was systematically analyzed by flow cytometry (FACSCalibur flow cytometer, BD Biosciences, San Jose, CA) using four-color combinations of monoclonal antibodies (mAbs) with fluorescein isothiocyanate (FITC), phycoerythrin (PE), allophycocyanin (APC), and phycoerythrin-cyanin 7 (PE-Cy7). Cell-Quest software (Becton Dickinson Biosciences) was used for data analysis. Monoclonal antibodies were purchased from the following manufacturers: BD Biosciences, CD1a-PE, CD5-FITC, CD7-FITC, CD10-APC, CD19-FITC, CD22-PE, CD23-PE, CD25-APC, CD33-FITC, CD34-PE, CD45-PE-Cy7, cyCD79a-PE, CD103-FITC, TCRγδ-PE, FMC7-FITC, HLA-DR-APC, surface immunoglobulin (sIg) M-PE, cytoplasmic immunoglobulin (cIg) M-APC; Beckman Coulter, CD3-APC, CD4-FITC, CD8-PE, CD11c-PE, CD13-PE, CD20-APC, CD117-PE, TCRαβ-FITC, sIg-Lamda-FITC, sIg-Kappa-APC.

At diagnosis, all samples were analyzed in two steps. Firstly, samples were stained with the following combinations of mAbs (CD7/CD117/HLA-DR/CD45, CD19/CD34/CD10/CD45, CD33/CD13/CD45), with CD45/side-scatter gating of blasts, to identify the basic immunophenotypic characteristics of blast cells: acute leukemia type (lymphoblastic or non-lymphoblastic) and lineage identity (B or T). Secondly, according to the ALL lineage, the following combinations of mAbs were used to further identify the immunophenotype of blast cells: CD5/CD22/CD20/CD45, CD103/CD11c/CD25/CD45, sIg Lamda/sIgM/sIg Kappa/CD45, FMC7/CD23/CD45, cIgM/cyCD79a/CD45 in B-lineage ALL; CD5/CD1a/CD20/CD45, TCRαβ/ TCRγδ/CD3/CD45, CD4/CD8/CD45 in T-lineage ALL.

For the investigation of MRD, the combination of mAbs was based on the aberrant phenotypes of leukemic blasts at diagnosis individually and at least 500 000 events were acquired. The MRD result was presented as the percentage of cells with aberrant phenotypes among nucleated cells. A sensitivity of 0.01% was achieved in all samples analyzed. Instrument setup was calibrated daily by analyzing Calibrite™ beads and standard blood sample (BD™ Multi-Check Control from BD Biosciences or CD-chex™ Plus from Streck, Inc.) for quality control.

**Fig 1:**
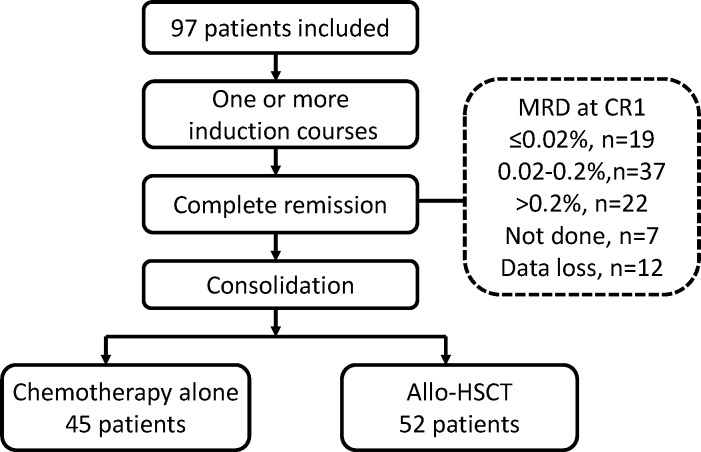
Patient Flow Diagram.

Bone marrow was collected at the end of induction for the evaluation of response. CR was defined as: (1) no circulating blasts or or extramedullary disease; (2) <5% bone marrow blasts by morphology with absolute neutrophil count ≥1,000/μL, and platelets ≥100,000/μL. Patients who failed the first induction would receive one or more different courses of induction until CR was obtained and bone marrow was collected after each course of induction. The number of induction courses to obtain CR was listed in Table 1. MRD was investigated at the time of first achievement of CR before consolidation, defined as MRD at CR1 (Fig 1). Nineteen samples of MRD at CR1 were not available in the analysis of MRD prognosis due to data loss (n = 7) or MRD not done (n = 12).

### Statistical analysis

Kaplan-Meier curve was used to estimate the probability of OS and leukemia free survival (LFS) and comparisons among subgroups were made by log-rank method. Cumulative incidences of relapse and non-relapse mortality (NRM) were determined by the competing risk method,[13] as described by Gooley.[14] Death in remission was a competing risk for relapse, and relapse was a competing risk for NRM. Receiver operating characteristic (ROC) analysis was used to get optimal cut-off points of MRD levels. Chi-square test and Fisher’s exact test were used to compare patient characteristics among subgroups. Multivariate analyses of variables affecting OS, LFS, and relapse rate were performed by Cox proportional-hazards regression model. All variables in the univariate analysis with a P-value at or below 0.1 were included in the multivariate analysis. A value of P<0.05 (two-sided) was considered statistically significant. Statistical analyses were performed in SPSS (Version 19.0) and R (http://www.r-project.org).

## Results

A total of 97 consecutive patients were retrospectively reviewed in this study. At the last follow-up, 49 patients had experienced a relapse (34 patients in the chemotherapy arm, 15 patients in the allo-HSCT arm), and 52 patients had died due to relapse or treatment-related mortality (31 patients in the chemotherapy arm, 21 patients in the allo-HSCT arm). The median follow-ups were 23 months (range, 3–81 months) for the entire cohort and 43 months (range, 11–81 months) for living patients. The 3-year OS and LFS rates for the whole cohort were 46.2% and 40.5%.

### Selection of cut-off points of MRD levels

ROC analysis was used to select the cut-off points of MRD at CR1 for OS in both the chemotherapy arm and the allo-HSCT arm. The MRD at CR1 level with the largest Youden index was selected as the cut-off point. In the chemotherapy arm, the area under curve (AUC) was 0.778 (95% confidence interval (CI) = 0.608–0.947, P = 0.007), and the cut-off point of MRD at CR1 was 0.0255%. In the allo-HSCT arm, the AUC was 0.740 (95% CI = 0.580–0.899, P = 0.009), and the cut-off point of MRD at CR1 was 0.2055%. Considering the tenfold relationship between these two cut-off points and also the convenience of data interpretation, we adopted 0.02% and 0.2% as the cut-off points of MRD levels for risk stratification. MRD≤0.02%, 0.02%<MRD≤0.2%, MRD>0.2% were defined as low, intermediate, and high MRD levels respectively.

### MRD in the chemotherapy arm

**Fig 2:**
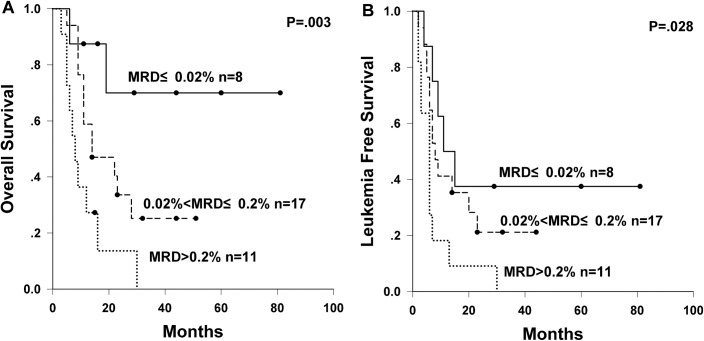
Survival of subgroups stratified by MRD at CR1 in the chemotherapy arm. (A) OS and (B) LFS of subgroups stratified by MRD at CR1 in the chemotherapy arm.

There were totally 36 evaluable samples of MRD at CR1 in the chemotherapy arm. Among all the clinical characteristics, only WBC count at diagnosis was unbalanced among MRD subgroups (low MRD subgroup vs. high MRD subgroup, P = 0.043). The 3-year incidence of relapse was significantly affected by MRD at CR1 level (low MRD subgroup 62.5% vs intermediate MRD subgroup 71.8% vs high MRD subgroup 90.9% at 3 years, P = 0.035), whereas the incidence of non-relapse mortality (NRM) was comparable among three MRD subgroups (P = 0.771). Furthermore, MRD at CR1 level had a significant impact on survival. The 3-year OS rates for low, intermediate, and high levels of MRD at CR1 were 70.0%, 25.2%, and 0%, respectively (P = 0.003, Fig 2A); and the 3-year LFS rates for low, intermediate, and high levels of MRD at CR1 were 37.5%, 21.2%, and 0%, respectively (P = 0.028, Fig 2B). MRD at CR1 and other risk factors (age, sex, WBC count at diagnosis, lineage, risk stratification, and induction courses before CR1) were subjected to univariate analysis for relapse, OS and LFS (S1 Table). MRD at CR1 and induction courses before CR1 with P≤0.1 in univariate analysis were further subjected to multivariate analysis for relapse, OS and LFS. In multivariate analysis (Table 2), higher level of MRD at CR1 had a significantly adverse effect on LFS (HR = 1.86, P = 0.030) and a trend towards higher relapse rate (HR = 1.72, P = 0.067). Both higher level of MRD at CR1 (HR = 2.67, P = 0.003) and ≥2 induction courses before CR1 (HR = 3.36, P = 0.019) were found to be significant risk factors for worse OS.

**Table 2:** Multivariate analysis for relapse, OS, and LFS in the chemotherapy arm.

Variables	Relapse			OS			LFS		
	HR	95%CI	P	HR	95%CI	P	HR	95%CI	P
**Induction courses before CR1**									
≥2 courses	2.161	0.846–5.520	0.107	3.361	1.224–9.229	0.019	2.200	0.865–5.596	0.098
1 course	1			1			1		
**MRD at CR1**									
Higher level of MRD	1.721	0.962–3.078	0.067	2.668	1.390–5.121	0.003	1.861	1.114–3.292	0.030
Lower level of MRD	1			1			1		

### MRD in the Allo-HSCT arm

**Fig 3:**
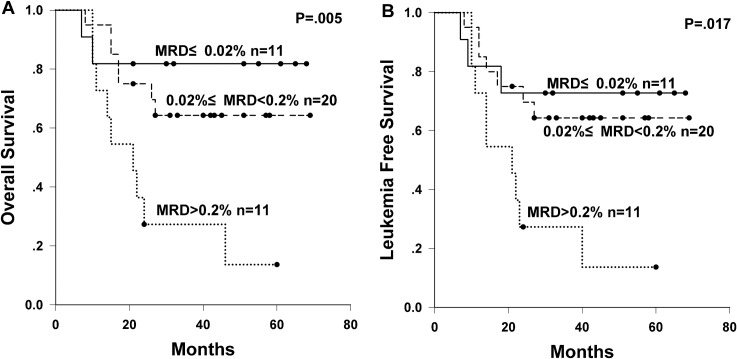
Survival of subgroups stratified by MRD at CR1 in the allo-HSCT arm. (A) OS and (B) LFS of subgroups stratified by MRD at CR1 in the allo-HSCT arm.

In total, 42 samples of MRD at CR1 were evaluable in the allo-HSCT arm. No significant difference of clinical characteristics was observed among MRD subgroups in comparison (P>0.05, details not shown). High MRD at CR1 subgroup tended to have a higher relapse rate compared with low and intermediate subgroups (high MRD subgroup 45.5% vs intermediate MRD subgroup 20.4% vs low MRD subgroup 18.2% at 3 years, P = 0.107); and the NRM rates were comparable among three subgroups (P = 0.510). MRD at CR1 was a strong prognostic factor for long term survival in the allo-HSCT arm. The 3-year OS rates for low, intermediate, and high levels of MRD at CR1 were 81.8%, 64.3%, and 27.3%, respectively (P = 0.005, Fig 3A); and the 3-year LFS rates were 72.7%, 64.3%, and 27.3%, respectively (P = 0.017, Fig 3B). MRD at CR1 and other risk factors (age, sex, WBC count at diagnosis, lineage, risk stratification, induction courses before CR1, and donor type) were subjected to univariate analysis for relapse, OS and LFS (S2 Table). MRD at CR1 was the only factor with P≤0.1 in univariate analysis for LFS (HR = 2.27, 95% CI = 1.14–4.53, P = 0.020). MRD at CR1 and lineage with P≤0.1 in univariate analysis were subjected into multivariate analysis for relapse and OS. Multivariate analysis (Table 3) confirmed that only higher level of MRD at CR1 was an independent adverse factor for OS (HR = 2.65, 95% CI = 1.23–5.69, P = 0.013), but failed to be significant in the multivariate analysis for relapse (P = 0.131).

**Table 3:** Multivariate analysis for relapse and OS in the allo-HSCT arm.

Variables	Relapse			OS		
	HR	95%CI	P	HR	95%CI	P
**B or T lineage**						
T	1.798	0.507–6.372	0.363	1.329	0.458–3.857	0.601
B	1			1		
**MRD at CR1**						
Higher level of MRD	2.054	0.806–5.233	0.131	2.646	1.230–5.692	0.013
Lower level of MRD	1			1		

### Chemotherapy versus Allo-HSCT by stratification of MRD at CR1

As the results shown above, MRD at CR1 was a prognostic factor for survival rates in both the chemotherapy arm and the allo-HSCT arm. Further, within the same levels of MRD at CR1, the differences of survival between the chemotherapy arm and the allo-HSCT arm were examined. Because of the retrospective nature of our analysis, a selection bias could not be excluded in comparison between these two arms since those patients who relapsed or died early after CR1 and had no chance to receive allo-HSCT were automatically allocated into the chemotherapy arm. Therefore, to decrease the selection bias, we did the landmark analysis. The median interval from CR1 to transplantation was 5 months (range 1.5–9 months) in the allo-HSCT arm, so patients in the chemotherapy arm relapsed or died in less than 5 months from CR1 were not included in this analysis. A total of 69 patients were included to the landmark analysis. The 3-year improved LFS rate with allo-HSCT was observed in intermediate MRD at CR1 subgroup (allo-HSCT 64.3% vs. chemotherapy 27.7%, P = 0.010) and high MRD at CR1 subgroup (allo-HSCT 27.3% vs. chemotherapy 0%, P = 0.022), but not in low MRD at CR1 subgroup (P = 0.206). The 3-year improved OS rate with allo-HSCT was only observed in intermediate MRD at CR1 subgroup (allo-HSCT 64.3% vs. chemotherapy 23.9%, P = 0.016), but not in low MRD at CR1 subgroup (P = 0.851) and high MRD at CR1 subgroup (P = 0.202).

### MRD at CR1 levels and conventional risk stratification

In total, 75 patients had both evaluable MRD at CR1 level and conventional risk stratification. The median levels of MRD at CR1 were 0.052% and 0.110% in patients of standard and high risk respectively, and there was no significant difference of MRD at CR1 level between two groups (P = 0.268, Mann-Whitney test), which indicated that there was no significant correlation between MRD at CR1 level and conventional risk stratification. The combination of MRD at CR1 level and conventional risk stratification could further define three risk groups. Standard risk patients with low level of MRD at CR1 formed low risk group which had an excellent survival with the 3-year OS rate being 100% and the 3-year LFS rate being 72.7%. Three out of 11 patients relapsed in this group. Two patients in the chemotherapy arm achieved a second remission after re-induction. One patient in the allo-HSCT arm experienced a CNS relapse 13 months after transplant and also achieved remission after several times of intrathecal chemotherapy. Conventional high risk patients with high level of MRD at CR1 were classified as the very-high-risk group with an extremely poor outcome. Both OS and LFS rates were only 10.0%. All of the four patients in the chemotherapy arm relapsed (at 1.5, 2.5, 3.5 and 5 months after reaching CR1) and died. In the allo-HSCT arm, only 1 patient was still alive without relapse, and the other 5 patients died at a median time of 6 months after transplant (4 patients died from relapse and 1 patient died from transplant-related mortality). The remaining patients presented the intermediate risk group with the 3-year OS rate of 43.0% and the 3-year LFS rate of 39.6%.

## Discussion

During the whole treatment cycle, there were different time points for MRD evaluation depending on different protocols, and the cut-off levels of MRD in different time points varied.[4–10] In this study, we chose the first achievement of CR as the MRD evaluation time point, which could be applied to most patients reaching CR no matter what induction protocols they received. MRD was detected by four-color flow cytometric immunophenotyping, of which sensitivity was lower than PCR analysis but the cost was relatively lower,[15] so it could be applicable in most developing regions. Considering the differences of treatment protocol and MRD-detection methodologies, we defined cut-off levels of MRD at CR1 using ROC analysis.

In the chemotherapy arm, MRD at CR1 was found to have a significantly negative correlation with the survival rate, which was in line with results from other MRD studies.[5–10] Higher level of MRD at CR1 predicted a higher relapse rate, which contributed to a lower survival rate. Almost all patients with high MRD at CR1 relapsed, and their median time between CR1 and relapse was only 6 months. Early intensified therapy should be considered for this subgroup of patients since the prognosis of relapsed ALL was dismal. The same prognostic effect of MRD at CR1 was observed in the allo-HSCT arm in our study (n = 42), and MRD at CR1 was an independent adverse factor for survival rate in the allo-HSCT arm. This relationship between early MRD level and posttransplantation outcome was also demonstrated in a prospective Northern Italy Leukemia Group trial, in which the outcome of allo-HSCT (n = 26) was sensibly affected by post-induction MRD level.[11] While Holowiecki et al [8] found no predictive value of MRD after induction in a cohort of patients receiving allo-HSCT (n = 35). However, it should be noted that this allo-HSCT cohort included only five patients with MRD≥0.1% compared with 30 cases with MRD<0.1% after induction. Larger prospective trials are warranted to further explore the relationship between early MRD level and clinical outcome in allo-HSCT since the sample sizes in each of the three studies are still relatively small.

For patients with positive MRD level, it was suggested that early intervention with intensified therapy including allo-HSCT could improve long-term survival. GMALL found that the 5-year OS was significantly higher for patients with molecular failure (MRD≥0.01%) and allo-HSCT in the first CR than for those without allo-HSCT in the first CR (54±8% vs. 33±7%; P = 0.06).[7] A recent GRALL study [16] showed that allo-HSCT was associated with longer RFS in patients with post-induction MRD≥0.1% (hazard ratio, 0.40) but not in good MRD responders. In our study, patients with intermediate and high level of MRD at CR1 (>0.02%) would benefit from allo-HSCT when compared with receiving chemotherapy alone. Allo-HSCT was recommended to be implemented in these MRD subgroups. But it should be noted, the prognosis for patients with high level of MRD at CR1 was dismal. Even with allo-HSCT, the survival rate was only 23.4%. Strategies to improve the clinical outcomes of this subgroup are urgently needed. On the other hand, patients with low level of MRD at CR1 (MRD≤0.02%) had an excellent clinical outcome whichever post-remission therapies they received. For patients in this subgroup, the selection of post-remission therapy was mainly up to the local experience and patients’ desire. Either chemotherapy or allo-HSCT would be suitable. All these results from different studies suggested that early MRD level was an excellent tool to decide post-remission therapy in adult ALL patients, and MRD evaluation should be incorporated into the routine treatment protocol. However, the cut-off point of MRD which was used for the decision of post-remission therapy varied across studies because of the differences in treatment protocols, patients’ background, and MRD-detection methodologies, etc. Further efforts should be taken to standardise the role of early MRD in formulating treatment decisions.

In summary, the results of our study suggest that MRD at CR1 was an independent prognostic factor for adult Ph-negative ALL in both chemotherapy alone and allo-HSCT arms, and the combination of MRD level with conventional risk criteria could further discriminate patients with different prognosis. When stratified by MRD at CR1, patients with intermediate and high levels of MRD at CR1 would benefit from allo-HSCT when compared with chemotherapy alone.

## Supporting Information

S1 Data Original data of all the included patients. (XLSX)

S1 Table Univariate analysis for relapse, OS, and LFS in the chemotherapy arm. (DOCX)

S2 Table Univariate analysis for relapse, OS, and LFS in the allo-HSCT arm. (DOCX)
