# Supplementary material for: Minimal Residual Disease at First Achievement of Complete Remission Predicts Outcome in Adult Patients with Philadelphia Chromosome-Negative Acute Lymphoblastic Leukemia
Source: PLoS One. 2016 Oct 3;11(10):e0163599. doi: 10.1371/journal.pone.0163599 (PMC5047486; doi:10.1371/journal.pone.0163599)
Supplement: S1 Table — (DOCX) [file pone.0163599.s002.docx]

| Variables | Relapse | | | OS | | | LFS | | |
| --- | --- | --- | --- | --- | --- | --- | --- | --- | --- |
|  | HR | 95%CI | P | HR | 95%CI | P | HR | 95%CI | P |
| **Age at diagnosis** |  |  |  |  |  |  |  |  |  |
| ≥35y | 0.885 | 0.412-1.902 | 0.755 | 0.643 | 0.276-1.494 | 0.304 | 0.816 | 0.383-1.739 | 0.598 |
| <35y | 1 |  |  | 1 |  |  | 1 |  |  |
| **Sex** |  |  |  |  |  |  |  |  |  |
| Female | 0.517 | 0.233-1.149 | 0.106 | 0.563 | 0.249-1.276 | 0.169 | 0.529 | 0.247-1.136 | 0.103 |
| Male | 1 |  |  | 1 |  |  | 1 |  |  |
| **WBC count at diagnosis** |  |  |  |  |  |  |  |  |  |
| ≥30×10^9^/L | 1.131 | 0.565-2.265 | 0.728 | 0.817 | 0.388-1.720 | 0.595 | 1.046 | 0.530-2.066 | 0.897 |
| <30×10^9^/L | 1 |  |  | 1 |  |  | 1 |  |  |
| **B or T lineage** |  |  |  |  |  |  |  |  |  |
| T | 1.362 | 0.632-2.935 | 0.430 | 1.189 | 0.531-2.664 | 0.674 | 1.299 | 0.607-2.780 | 0.500 |
| B | 1 |  |  | 1 |  |  | 1 |  |  |
| **Risk stratification** |  |  |  |  |  |  |  |  |  |
| High | 1.526 | 0.757-3.078 | 0.237 | 1.417 | 0.673-2.984 | 0.359 | 1.444 | 0.725-2.880 | 0.296 |
| Standard | 1 |  |  | 1 |  |  | 1 |  |  |
| **Induction courses before CR1** |  |  |  |  |  |  |  |  |  |
| ≥2 courses | 2.335 | 1.000-5.452 | 0.050 | 2.753 | 1.173-6.459 | 0.020 | 2.368 | 1.015-5.525 | 0.046 |
| 1 course | 1 |  |  | 1 |  |  | 1 |  |  |
| **MRD at CR1** |  |  |  |  |  |  |  |  |  |
| Higher level of MRD | 1.789 | 1.022-3.134 | 0.042 | 2.710 | 1.445-5.083 | 0.002 | 1.915 | 1.114-3.292 | 0.019 |
| Lower level of MRD | 1 |  |  | 1 |  |  | 1 |  |  |

**S1 Table. Univariate analysis for relapse, OS, and LFS in the chemotherapy arm.**
