# Supplementary material for: Minimal Residual Disease at First Achievement of Complete Remission Predicts Outcome in Adult Patients with Philadelphia Chromosome-Negative Acute Lymphoblastic Leukemia
Source: PLoS One. 2016 Oct 3;11(10):e0163599. doi: 10.1371/journal.pone.0163599 (PMC5047486; doi:10.1371/journal.pone.0163599)
Supplement: S2 Table — (DOCX) [file pone.0163599.s003.docx]

**S2 Table. Univariate analysis for relapse, OS, and LFS in the allo-HSCT arm.**

| Variables | Relapse | | | OS | | | LFS | | |
| --- | --- | --- | --- | --- | --- | --- | --- | --- | --- |
|  | HR | 95%CI | P | HR | 95%CI | P | HR | 95%CI | P |
| **Age at diagnosis** |  |  |  |  |  |  |  |  |  |
| ≥35y | 1.264 | 0.401-3.982 | 0.689 | 1.092 | 0.399-2.988 | 0.863 | 1.305 | 0.510-3.343 | 0.579 |
| <35y | 1 |  |  | 1 |  |  | 1 |  |  |
| **Sex** |  |  |  |  |  |  |  |  |  |
| Female | 1.332 | 0.482-3.682 | 0.580 | 0.859 | 0.361-2.042 | 0.731 | 0.983 | 0.424-2.278 | 0.967 |
| Male | 1 |  |  | 1 |  |  | 1 |  |  |
| **WBC count at diagnosis** |  |  |  |  |  |  |  |  |  |
| ≥30×10^9^/L | 2.139 | 0.747-6.120 | 0.156 | 1.351 | 0.551-3.312 | 0.511 | 1.310 | 0.542-3.168 | 0.549 |
| <30×10^9^/L | 1 |  |  | 1 |  |  | 1 |  |  |
| **B or T lineage** |  |  |  |  |  |  |  |  |  |
| T | 2.778 | 1.005-7.681 | 0.049 | 2.116 | 0.870-5.147 | 0.099 | 1.835 | 0.768-4.386 | 0.172 |
| B | 1 |  |  | 1 |  |  | 1 |  |  |
| **Risk stratification** |  |  |  |  |  |  |  |  |  |
| High | 1.702 | 0.590-4.910 | 0.325 | 1.588 | 0.657-3.836 | 0.304 | 1.400 | 0.594-3.300 | 0.441 |
| Standard | 1 |  |  | 1 |  |  | 1 |  |  |
| **Induction courses before CR1** |  |  |  |  |  |  |  |  |  |
| 2 courses | 1.539 | 0.433-5.469 | 0.505 | 1.595 | 0.533-4.774 | 0.404 | 1.378 | 0.466-4.080 | 0.562 |
| 1 course≥ | 1 |  |  | 1 |  |  | 1 |  |  |
| **Donor types** |  |  |  |  |  |  |  |  |  |
| Unrelated | 0.981 | 0.287-3.352 | 0.975 | 1.908 | 0.525-6.935 | 0.327 | 1.400 | 0.439-4.466 | 0.570 |
| Haploidentical | 0.826 | 0.206-3.307 | 0.788 | 2.245 | 0.593-8.494 | 0.234 | 1.639 | 0.493-5.446 | 0.420 |
| HLA-matched sibling | 1 |  |  | 1 |  |  | 1 |  |  |
| **MRD at CR1** |  |  |  |  |  |  |  |  |  |
| Higher level of MRD | 2.415 | 1.007-5.793 | 0.048 | 2.817 | 1.357-5.848 | 0.005 | 2.273 | 1.139-4.534 | 0.020 |
| Lower level of MRD | 1 |  |  | 1 |  |  | 1 |  |  |
